# A Scoping Review of Non-invasive Imaging Modalities in Dermatological Disease: Potential Novel Biomarkers in Hidradenitis Suppurativa

**DOI:** 10.3389/fmed.2019.00253

**Published:** 2019-11-06

**Authors:** David Grand, Kristina Navrazhina, John W. Frew

**Affiliations:** ^1^Laboratory of Investigative Dermatology, The Rockefeller University, New York, NY, United States; ^2^Albert Einstein College of Medicine, Bronx, NY, United States; ^3^Weill Cornell/Rockefeller/Sloan Kettering Tri-Institutional MD-PhD Program, Weill Cornell University, New York, NY, United States

**Keywords:** hidradenitis suppurativa, acne inversa, ultrasound, non-invasive, confocal microscopy, electrical impedance, MRI

## Abstract

**Background:** The development of imaging-based biomarkers has the potential to overcome major challenges in the accurate and reproducible assessment of disease severity and response to novel therapies in Hidradenitis Suppurativa (HS). Understanding the advantages and limitations of existing non-invasive imaging modalities in dermatological disease will aid in the development of hypotheses and inform the design of future studies.

**Methods:** A scoping review was performed using Medline, Embase, Web of Science Databases and evaluation of “gray literature” until June 30, 2019. Citations were examined according to pre-defined inclusion and exclusion criteria. Citations were reviewed by two independent reviewers. Narrative Synthesis was used to summarize data, structured by imaging modality.

**Results:** Non-invasive imaging modalities, such as ultrasound, MRI, RCM, EIS, OCT, and MIT, were identified. Only ultrasound, MRI and MIT have been used in HS. Image modalities vary in image depth, resolution, cost, accessibility and correlation with known aspects of disease activity in HS.

**Discussion and Conclusion:** The benefits and limitations of each imaging modality are products of cost, accessibility, validity and reliability. An additional hurdle to the development of image-based biomarkers in HS is a lack of established analytical benchmarks that can be correlated with existing biological, inflammatory and clinical parameters. This review has identified potential imaging biomarkers, as well as relevant analytical benchmarks that reflect the presence or absence of disease. Further investigation work is needed to analytically and clinically validate these imaging variables in order to identify potential imaging biomarkers in HS.

## Introduction

The degree of interest in non-invasive imaging modalities in dermatology has increased dramatically over the last decade. Given that visual inspection alone is limited in evaluating disease extent, histopathological assessment of skin through the use of biopsies remains a vital part of a dermatologist's armamentarium (1). Non-invasive imaging modalities have the potential to compliment, and in some instances, replace, the need for skin biopsies. Although this prospect is promising, it is dependent upon whether the specific aspect of the image, known as the imaging biomarker, is both analytically and clinically valid (through assessment of sensitivity, specificity, accuracy, precision, and other relevant performance characteristics against a gold standard test and a pre-defined clinical endpoint) (2) and whether the process of obtaining the imaging biomarker is cost effective and easily administered (3). The field of oncology demonstrates successful use of imaging biomarkers in diagnosis and therapeutic monitoring (3). Well-established guidelines exist for the development and validation of such biomarkers. Imaging-based biomarkers are desirable for diseases that demonstrate the following qualities: a disconnect between clinical examination and the disease status of the patient; a lack of a validated, reliable blood or serum biomarker; and a cost-effective reproducible method of imaging, the results of which will provide a clinical benefit to the patient at a pre-defined endpoint.

Hidradenitis Suppurativa (HS) is a chronic inflammatory disorder which is often clinically underestimated in terms of disease extent, thereby necessitating the use of imaging modalities such as ultrasound to map disease extent. Validated and/or reliable blood/serum-based biomarkers do not currently exist. The identification, development and validation of imaging-based biomarkers in HS has the potential to improve diagnostic and disease severity assessments. If validated against pre-defined disease characteristics, these imaging modalities have the potential to be developed into therapeutic and predictive biomarkers of clinical response. This is particularly desirable given issues with poor reliability of clinical assessments (4) and elevated placebo response rates in clinical trials (5). Whilst many tests are routinely utilized as diagnostic biomarkers in other areas of medicine (e.g., prostate-specific antigen) and predictive/safety biomarkers have been routinely used in dermatology (e.g., TPMT polymorphisms prior to Azathioprine therapy and G6PD polymorphisms prior to Dapsone therapy) (6), it is unclear which aspects of non-invasive imaging modalities have been identified and/or analyzed for their potential as biomarkers of dermatologic disease.

### Aims

We undertook a scoping review of the literature pertaining to non-invasive imaging modalities in dermatologic disease with a particular focus on aspects of imaging that may be applicable to HS. The aim of a scoping review is to “*map the body of literature pertaining to a research question*” (7). Scoping reviews are particularly useful in emerging fields where the aim is to “*identify knowledge gaps, scope a body of literature or…clarify concepts*” (8). They differ from systematic reviews in that the overall aims of the review encompass a broader field of inquiry, whilst still adhering to rigorous pre-defined methodology (8). The outcomes of a scoping review may be used to inform a further systematic review, or develop concepts amenable to further research and investigation.

### Methods

This scoping review was developed using previously described methodological framework of Peters et al. (9) Literature search was undertaken to identify papers regarding non-invasive imaging modalities in dermatologic disease. A particular focus was made on inflammatory dermatoses, particularly on HS. Inclusion criteria were all study designs, any language, any imaging technique, all age groups, sex, ethnicity, healthcare settings, and treatment modalities. Exclusion criteria included animal studies, and *ex vivo* imaging studies.

### Search Strategy

Databases searched included MEDLINE (1946–June 30, 2019), Embase (1980–June 30, 2019) and Web of Science (1990–June 30, 2019). Gray literature was searched using Google Scholar and the first 100 results to the developed search strategy. Terms included in the search strategy included (skin) AND (imaging OR ultrasound OR MRI OR impedance OR confocal) AND (*in vivo*) AND (diagnosis OR biomarker) AND (inflammation).

### Study Selection

All identified citations from databases (*n* = 884) and the first 100 citations in the gray literature (*n* = 100) were manually examined, and duplicates removed by one author (DG). Two authors (DG and JF) independently reviewed the titles and abstracts from the remaining papers (*n* = 372). All papers referring to non-human studies were excluded (*n* = 169). The remaining articles (*n* = 203) were screened through examination of the full text, with an additional 47 articles excluded as not pertaining to dermatological disorders. The inclusion or exclusion of citations was based upon unanimous agreement by the two independent reviewers with disagreements mediated by referral to a third independent reviewer for mediation (KN). Citations were categorized according to type of imaging modality employed.

### Data Extraction

Data was extracted by two independent reviewers (DG and JF) with data collated and presented as a narrative synthesis categorized by imaging modality.

## Results

**Figure 1:**
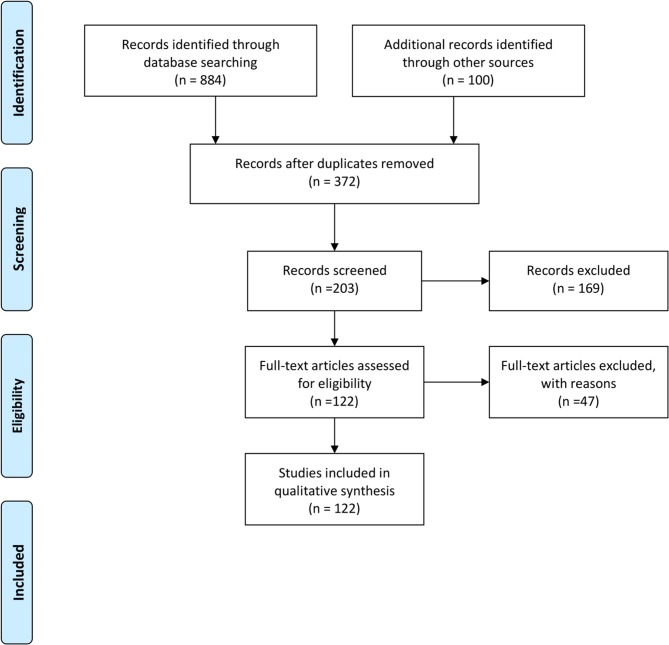
PRISMA diagram.

A total of 122 manuscripts were included in review. A PRISMA diagram outlining the literature review process is presented in Figure 1. Six main imaging modalities were identified: Ultrasound, Magnetic resonance imaging (MRI), Reflectance Confocal Microscopy (RCM), Electrical Impedance Spectroscopy (EIS), Optical Coherence Tomography (OCT), and Medical Imaging Thermography (MIT).

### Ultrasound

Ultrasound is an affordable and portable method of imaging both epidermal characteristics such as epidermal thickness (10, 11) and deeper subcutaneous structures (10, 12). Typical ultrasound probe frequencies used for cutaneous examination range from 7.5 to 20 MHz (11). However, higher frequency probes (50–150 MHz) can allow for more accurate representation of epidermal thickness as seen in psoriasis (12–14). The limitations of such high frequency probes are reduced penetration depth and hence lower resolution regarding deeper structures (15–17), hence a combination of probe frequencies often present the best option for multiple targets in cutaneous tissues. Epidermal measurements <0.1 mm are prone to inaccuracy using Ultrasound (10).

Ultrasound has been used to quantify epidermal thickness in lichen planus and psoriasis (15, 16). Epidermal thickness, as measured by ultrasound, demonstrates correlation with the maximum depth of rete ridges in histological specimens (17). Alterations in vessel distribution, as measured by Doppler ultrasound (18), have been correlated with metastatic potential of cutaneous malignancies (BCC, SCC, Melanoma) (19). Dermal fibrosis and adipose tissue alterations are visible and have been documented in HS, scleroderma and graft-vs.-host-disease (20, 21). Although color Doppler is able to provide quantitative data regarding flow rates in non-dermatological conditions [for example, echocardiography and tumor blood flow (22, 23)], such data is not widely validated in cutaneous inflammation with histological specimens. Additionally, Doppler ultrasound requires an informed choice of transducer and appropriate technical training in order to optimize for each patient and standardize for each visit (24). Therefore, a combination of probe frequencies allows for examining multiple targets in cutaneous tissues.

In normal tissues, the epidermis appears as a thin, hyperechoic line, whereas the dermis appears as a broad, echogenic band. Fat in the subcutaneous tissue appears hypoechoic while the surrounding fascia and connective tissue is hyperechoic (21). Sonographic studies in HS patients identified alterations in epidermal thickness (25) as broadly correlating to the well documented psoriasiform epidermal hyperplasia seen in histological sections (26). Diffuse dermal edema and inflammatory infiltrates also correspond to heterogenous hyperechoic dermal alterations in the reticular dermis. (25). Scoring systems have been proposed based upon qualitative sonographic descriptions (27) which correlate with HS disease activity, quality of life and pain scores (28, 29). This can be explained by the fact that visual inspection of HS lesional skin alone often underestimates the true extent of disease (30, 31). This fact justifies the use of sonography to define disease extent preoperatively (32).

Potential quantitative biomarkers that may be derived from ultrasound data include epidermal thickness, tunnel diameter, Doppler flow in dermal vessels and adjacent to structures including subcutaneous fluid collections. Given the well understood mechanism of Th17 immunologic axis activation and psoriasiform epidermal hyperplasia (33, 34), proof of concept studies into the correlation between epidermal thickness as measured by ultrasound and tissue cytokine level (such as IL-23, IL-17A, IL-17F) would aid in establishing its utility as an indirect biomarker of Th17 activity.

### Magnetic Resonance Imaging (MRI)

MRI produces high resolution images of deep subcutaneous structures with specific highlighting of layers of fat-containing (T1-weighted) and water-containing (T2-weighted) content (10, 35–37). Relatively little resolution of the epidermis and dermis is seen (10), although ultrasensitive radiofrequency detectors can provide additional resolution to more superficial structures (38). Given its relatively high cost and time consumption compared to ultrasound, it is rarely utilized in dermatologic practice and research (13). The utility of MRI in dermatology is in the assessment of deep involvement of subcutaneous, fascial and muscular structures in inflammatory disorders such as morphea and sclerodermoid disorders (35), as well as in management of patients with inherited disorders such as neurofibromatosis (36) and vascular and lymphatic malformations (37).

MRI has been employed as a diagnostic modality to differentiate HS tunnels from peri-anal Crohn's disease and enterocutaneous fistulae, as well as being a pre-operative assessment tool prior to wide surgical excision of HS lesions (39). T2-enhancing cutaneous thickening, subcutaneous fat stranding, rim-enhancing abscesses, sinus tracts, and lymphadenopathy have been documented in the setting of HS (40). Structural changes (presence and dilation of dermal tunnels, number of abscesses) were documented over time suggesting a degree of sensitivity that could be used to track the progression of disease and relationship to subjective patient symptomatology (41). No documented use of radiofrequency detectors have been identified in cutaneous disease.

Given the benefits in high resolution identification of structural abnormalities in deeper tissues (10), MRI's major benefits relate to accurate identification of the disease extent, particularly in the peri-operative setting. Cost, time and the need for specialized equipment may limit the widespread adoption of MRI in the routine evaluation of HS (13). Given the documented alterations in dermal tunnel size in longitudinal imaging studies (27), dilation of dermal tunnels may have potential as an indirect marker of tunnel inflammatory activity and drainage. As current assessment outcomes (HiSCR, IHS4, AISI) (42–44) measure only the presence or absence of tunnel drainage, imaging modalities such as MRI may provide surrogate outcomes (such as tunnel diameter) which may relate to the degree of drainage. Validation of these measures could be made against pre-existing wound exudate measures and estimated volumes of discharge (based on dressing weights) in HS.

### Reflectance Confocal Microscopy (RCM)

RCM provides high resolution cellular imaging and is commonly employed in the assessment and monitoring of melanocytic neoplasms (45). Relative to other imaging modalities, it is low cost. Images in the horizontal (x-axis) and vertical (y-axis) planes provide an “en face” view (45). Z-axis views can be reconstructed but the field of view is limited by a penetration depth of ~200 μm. This roughly corresponds to the papillary dermis, limiting the utility of RCM to structural and morphological alterations of the epidermis and papillary dermis such as vascular density (12, 13). Additional limitations to RCM are largely dependent upon operator experience (46).

The use of RCM has been not reported in HS lesional skin; however, it has been used to identify morphological and vascular changes in other inflammatory dermatoses. Increased dermal papillary cross-sectional area, increased papillary capillary density and the presence of Munro abscesses have been shown to evolve with the clinical progression and resolution of psoriasis (47, 48). Comedonal structures have been characterized by RCM in acne vulgaris, as well as features preceding clinical resolution with the use of topical acne therapies (49, 50). Langerhan's cell and dermal dendritic cells are well visualized with RCM (51), and density variations have been noted in apocrine gland rich skin of normal individuals compared with apocrine gland poor skin (26). Psoriasiform epidermal hyperplasia (26), comedones (52) and increased dendritic cell concentrations (26) have all been identified histologically in HS lesional tissue, so examination of the morphological characteristics of HS lesional tissue using RCM could be correlated with these histological and other investigative findings in the future in order to ascertain if RCM has the potential to identify prospective imaging biomarkers.

### Electrical Impedance Spectroscopy (EIS)

Electrical Impedance Spectroscopy (EIS) is a measurement of tissue bioimpedance. Whilst it does not produce an “image” in the traditional sense of ultrasound or MRI, it is an emerging potentially important non-invasive assessment modality. Bioimpedance reflects cellular size, shape, orientation, compaction, and cell membrane structure, and is able to reliably distinguish variations between healthy and diseased tissue (53). Currently, EIS is used as an adjuvant diagnostic tool in the setting of melanocytic lesions, producing detailed bioimpedance data which is summarized as a score from 0 to 10. Higher scores indicate altered bioimpedance compared to within-patient control sites (11). EIS led to a change in a decision to biopsy lesions in 25% of cases, improved diagnostic accuracy, led to fewer biopsies of benign lesions and more biopsies of malignant lesions (54). It is currently unclear if EIS demonstrates altered signals in lesional HS tissue. However, given the known association of altered bioimpedance with tissue water content (55), systemic inflammation (56), and tissue catabolism in diabetes (57), it can be hypothesized that bioimpedance may be altered in HS lesional tissue due to a combination of local tissue and systemic factors. One should note a major limitation of bioimpedance measurements in general is an assumed constant hydration factor (58). Although this is partially addressed through the use of saline swabs prior to impedance measurements, formal evaluation of reliability and accuracy in the setting of inflammatory dermatoses (as opposed to melanocytic lesions) is required. Systematic analytical validation of EIS results in the setting of inflammatory dermatoses would be required prior to definitive statements regarding potential utility in HS.

### Optical Coherence Tomography (OCT)

Optical coherence tomography (OCT) is an interferometric imaging procedure that utilizes the scattering of reflected light to generate a three-dimensional image. It labels the epidermis (particularly in glabrous skin) with high contrast as well as differentiates between papillary and reticular dermis. OCT penetrates to a depth of 1–1.5 mm and can be viewed en-face or as reconstructed three-dimensional images (12, 59). OCT accurately distinguishes actinic keratosis from basal cell carcinoma (60) and quantifies psoriasiform epidermal thickening and dermal vascular dilatation in psoriasis (61). OCT has also quantified changes in blood flow in response to topical treatment with brimonidine gel in rosacea (62). The cost of equipment is a potential barrier to widespread adoption of OCT; however, it has the potential for increased accuracy and resolution of epidermal and dermal structures compared with ultrasound. Other potential limitations include the effect of subcutaneous abscesses and scarring in HS upon the accuracy of its readings. Vitreal hemorrhage and exudate can obscure retinal OCT imaging (63) and the presence of fibrotic tissue resulted in false positive readings suggestive of basal cell carcinoma in healthy tissue (64).

### Medical Infrared Thermography (MIT)

Medical infrared thermography (MIT) detects minimal temperature differences between different skin areas based on infrared emissions. It produces a two-dimensional thermal map in which brightness correlates with tissue temperature down to a depth of 2–3 mm (65). MIT has assessed burn wound depth (66) and is being actively investigated as an alternative to ultrasound in the grading of HS. MIT has been correlated with qualitative changes in Doppler blood flow in HS (67) and have defined tissue margins in HS skin removal surgery (67, 68). It remains unclear whether the accuracy and reproducibility of MIT is influenced by body site, previous surgical excisions and microvascular disease from smoking and/or metabolic syndrome, which may all influence tissue blood flow (69). MIT has shown to have significant variability between sessions and subjects (70), bringing into question the utility of MIT as a longitudinal assessment modality.

#### Identifying Analytical and Clinical Benchmarks for Future Biomarker Validation

The results of this scoping review have identified a number of imaging outcomes that have potential to be assessed for analytical and clinical validity in the setting of inflammatory skin diseases such as HS (Table 1). Currently little is known regarding the correlation of imaging findings in HS with known biological markers of disease activity and severity. In lesional and non-lesional tissue, quantification of TNF-α, IL-17, and IL-1β mRNA has correlated with HS disease severity (71, 72) and response to treatment (34). This raises the possibility that lesional skin mRNA levels can be used as an analytical standard from which to assess the validity of imaging biomarkers. Given the well-established role of inflammation (particularly IL-17) in epidermal changes, including psoriasiform hyperplasia (73), it is reasonable to hypothesize that non-invasive imaging findings would parallel biological and clinical response, albeit in a delayed fashion compared with mRNA and immunohistochemistry. In order to extrapolate imaging biomarkers into surrogate outcomes of clinical response, longitudinal studies and reliable clinical outcome benchmarks are required (2). Given the known variability in HS clinical rating scales, multiple clinical outcome scales may need to be utilized. The need for accurate longitudinal measurements implies that MIT may not be an appropriate imaging modality to develop as a surrogate outcome of clinical response (4).

**Table 1:** Potential imaging-based biomarkers identified for further investigation from this scoping review.

**Imaging modality**	**Potential imaging biomarker**	**Potential disease activity correlate(s)**	**Potential clinical correlate(s)**
Ultrasound	Epidermal Thickness	IL-23, IL-22, IL-17A, IL-17C, TNF-alpha, Histologic sections	HiSCR, IHS4, AISI,
	Dermal/Hypodermal Vascularity	VEGF, TNF-alpha, Histologic sections	HiSCR, IHS4, AISI, pain score
	Dermal/hypodermal Tunnel Diameter	Histologic sections	Volume of tunnel drainage, IHS4, Pain Score
	Vascularity adjacent to subcutaneous collections	VEGF, TNF-alpha, Histologic sections	HiSCR, IHS4, AISI, pain score
MRI	Dermal/Hypodermal Tunnel Diameter	Histologic sections	Volume of tunnel drainage, IHS4, Pain score
RCM	Dendritic Cell Density	Histologic sections, CD1a, CD207,	HiSCR, IHS4, AISI,
	Epidermal Thickness	IL-23, IL-22, IL-17A, IL-17C, histologic sections	HiSCR, IHS4, AISI,
	Dermal Papillae Vascularity	VEGF, TNF-alpha, Histologic sections	HiSCR, IHS4, AISI, Pain score
OCT	Epidermal Thickness	IL-23, IL-22, IL-17A, IL-17C, Histologic sections	HiSCR, IHS4, AISI,
	Dermal Fibrosis	Histologic sections	HiSCR, IHS4, AISI,
	Dermal Vascularity	VEGF, TNF-alpha, histologic sections	HiSCR, IHS4, AISI, Pain score

## Future Directions

Currently, ultrasound is the most widely used imaging modality in clinical practice for HS. The low cost and accessibility are aspects that make ultrasound-based imaging biomarkers attractive for future validation. Emerging methods such as OCT and RCM demonstrate higher degrees of accuracy regarding epidermal thickness which may outweigh the great cost. The utility of MRI is largely restricted to pre-operative disease assessment; however, it is the only modality with a proof of concept to be able to measure deep dermal tunnel dilatation as a quantifiable surrogate measure of tunnel drainage. EIS has a number of potential confounding effects that require exploration in the setting of inflammatory skin disease, and MIT is limited by its lack of reproducibility. In summary, we have identified a number of potential imaging-based variables which require further investigation to evaluate their potential as imaging-based biomarkers for HS and discussed their benefits and limitations pertaining to their potential to meet criteria for biomarker validity and reliability.

## Author Contributions

DG, KN, and JF were responsible for the concept of the study. DG wrote the first draft of the manuscript. JF and KN wrote portions of the manuscript. All authors read and approved of the final version of the manuscript.

### Conflict of Interest

The authors declare that the research was conducted in the absence of any commercial or financial relationships that could be construed as a potential conflict of interest.
